# 466. All-Cause Mortality in Patients with Invasive Candidiasis or Candidemia from an Interim Analysis of a Phase 3 Open-label Study (FURI)

**DOI:** 10.1093/ofid/ofac492.524

**Published:** 2022-12-15

**Authors:** Juergen Prattes, Thomas King, Nkechi Azie, David A Angulo

**Affiliations:** Medical University of Graz, Graz, Steiermark, Austria; SCYNEXIS, Inc., Jersey City, New Jersey; SCYNEXIS, Inc., Jersey City, New Jersey; SCYNEXIS, Inc., Jersey City, New Jersey

## Abstract

**Background:**

There are limited oral antifungal treatment options for patients with high-mortality fungal infections such as candidemia or invasive candidiasis who fail available antifungals or have infection caused by resistant fungi. Ibrexafungerp is a broad-spectrum glucan synthase inhibitor with activity against *Candida* spp., including azole- and echinocandin-resistant strains. A Phase 3 open-label study of ibrexafungerp (FURI; NCT03059992) is ongoing for patients intolerant of, or invasive fungal disease refractory to, standard antifungal therapy. We present an interim analysis of all-cause mortality (ACM) through 30 d post-treatment for patients with candidemia or invasive candidiasis (IC) who completed therapy up until Oct 2020.

**Methods:**

FURI patients are eligible for enrollment with proven/probable severe mucocutaneous candidiasis or IC, or candidemia, with documented evidence of failure, intolerance, or toxicity related to an approved standard-of-care antifungal treatment; or patients who cannot receive oral antifungals (e.g., due to susceptibility), and continued IV antifungal therapy is clinically undesirable/unfeasible. Patients were followed through 30 d post-treatment for ACM.

**Results:**

Out of the 74 patients who completed therapy in the FURI study through October 2020, 39 (52.7%) had IC or candidemia and received ibrexafungerp. The most common infections in this group were candidemia (28.2%), intra-abdominal infection (28.2%), and bone infection (20.5%).

Survival at ≥ 30 d post-treatment was 92.3%. Three patients (7.7%) died within 30 d post ibrexafungerp, a 4th died at 31 d, and a 5th died at 56 d. The mean age of the expired patients was 54 yr. Three patients had candidemia (2 with *C. parapsilosis* and 1 with *C. albicans*), and 2 had intra-abdominal candidiasis, (both with *C. glabrata*). The average time on ibrexafungerp was 16.6 d. The mean time to death post-treatment for these patients was 22 d (median 8 d). In each case, the deaths were due to causes other than the underlying fungal disease.

**Conclusion:**

Analysis of all-cause mortality in these patients from the FURI study indicates that oral ibrexafungerp provides a favorable therapeutic response in patients with challenging fungal disease and limited treatment options.

**Disclosures:**

**Thomas King, MS MPH**, SCYNEXIS, Inc.: employee|SCYNEXIS, Inc.: Stocks/Bonds **Nkechi Azie, MD MBA FIDSA**, SCYNEXIS, Inc.: employee|SCYNEXIS, Inc.: Stocks/Bonds **David A. Angulo, MD**, SCYNEXIS, Inc.: employee|SCYNEXIS, Inc.: Stocks/Bonds.

